# Medications and patient safety in the trauma setting: a systematic review

**DOI:** 10.1186/s13017-019-0225-6

**Published:** 2019-02-15

**Authors:** Jonathan H. DeAntonio, Tammy Nguyen, Gregory Chenault, Michel B. Aboutanos, Rahul J. Anand, Paula Ferrada, Stephanie Goldberg, Stefan W. Leichtle, Levi D. Procter, Edgar B. Rodas, Alan P. Rossi, James F. Whelan, V. Ramana Feeser, Michael J. Vitto, Beth Broering, Sarah Hobgood, Martin Mangino, Dayanjan S. Wijesinghe, Sudha Jayaraman

**Affiliations:** 1 Division of Acute Care Surgery, Department of Surgery, Virginia Commonwealth University, VCU Health, Richmond, Virginia USA; 2 Department of Emergency Medicine, Virginia Commonwealth University, VCU Health, Richmond, Virginia USA; 3 VCU Health Department of Pharmacy Services, Critical Care, Richmond, Virginia USA; 4 Program for Global Surgery, Department of Surgery, Virginia Commonwealth University, VCU Health, Richmond, Virginia USA; 5 Division of Geriatrics, Department of Internal Medicine, Virginia Commonwealth University, VCU Health, Richmond, Virginia USA; 6 Department of Pharmacotherapy and Outcomes Sciences and Laboratory of Pharmacometabolomics and Companion Diagnostics, Virginia Commonwealth University School of Pharmacy, VCU Health, Richmond, Virginia USA; 7 Department of Surgery, VCU School of Medicine, VCU Health System, Virginia Commonwealth University, Richmond, Virginia USA; 8 VCU School of Medicine, Richmond, Virginia USA

**Keywords:** Trauma medication reconciliation, Med rec, Medication error

## Abstract

**Background:**

Medication errors account for the most common adverse events and a significant cause of mortality in the USA. The Joint Commission has required medication reconciliation since 2006. We aimed to survey the literature and determine the challenges and effectiveness of medication reconciliation in the trauma patient population.

**Materials and methods:**

We conducted a systematic review of the literature to determine the effectiveness of medication reconciliation in trauma patients. English language articles were retrieved from PubMed/Medline, CINAHL, and Cochrane Review databases with search terms “trauma OR injury, AND medication reconciliation OR med rec OR med rek, AND effectiveness OR errors OR intervention OR improvements.”

**Results:**

The search resulted in 82 articles. After screening for relevance and duplicates, the 43 remaining were further reviewed, and only four articles, which presented results on medication reconciliation in 3041 trauma patients, were included. Two were retrospective and two were prospective. Two showed only 4% accuracy at time of admission with 48% of medication reconciliations having at least one medication discrepancy. There were major differences across the studies prohibiting comparative statistical analysis.

**Conclusions:**

Trauma medication reconciliation is important because of the potential for adverse outcomes given the emergent nature of the illness. The few articles published at this time on medication reconciliation in trauma suggest poor accuracy. Numerous strategies have been implemented in general medicine to improve its accuracy, but these have not yet been studied in trauma. This topic is an important but unrecognized area of research in this field.

## Background

The Institute of Medicine’s (IOM), *To Err is Human: Building a Safer Health System* (1999), raised awareness of medication errors in health care [1]. Medication errors are a frequent cause of adverse events and result in substantial mortality in the USA [2, 3]. Medication lists are often inaccurate across outpatient, emergency department, inpatient, and discharge settings [4–8]. Fifty percent of medication errors occur at admission, transitions of care, and discharge [4]. Adverse drug events increase the length of stay by 2.2 days and at a cost of $2595 per event at a minimum [9].

Recognizing this, a second IOM report, *Preventing Medication Errors*, aimed to reduce medication errors and improve patient safety by emphasizing the importance of medication reconciliation [10]. Medication reconciliation involves obtaining an accurate list of medications, including dosage, route of intake, frequency, and adherence and then updating the medical record with these medications [11]. The Joint Commission on Accreditation of Healthcare Organizations (JCAHO) mandated medication reconciliation throughout the continuum of healthcare in their 2006 National Patient Safety Goals, but it can be difficult, time-consuming, inaccurate, and costly [12–15]. An admission medication reconciliation requires time—on average 58.4 min with a cooperative patient—and costs a minimum of $55.91 per patient for a pharmacist to complete [13]. Other challenges include health illiteracy, lack of appropriate staffing, complex medication names and regimens, and polypharmacy with increasing age [16–19]. The attribution and impact of medication errors as well as prevention of errors using current medication reconciliation can be difficult to determine [4, 18, 20, 21].

These important challenges are only that much more profound in the emergent setting. Trauma is the number one cause of death below the age of 45 in the USA and results in $600 billion in annual costs [22]. Based on the Center for Disease Control, there are 130 million emergency visits with 37 million trauma visits in the USA on an annual basis. Trauma patients have unique problems which prevent accurate medication reconciliation—severe or distracting injuries, alterations of consciousness, intoxication, anxiety associated with trauma, and the urgent nature of emergency care. These limitations put this patient population at even higher risk of complications compared to general medical patients.

We aimed to perform a systematic review to evaluate the existing literature for articles on medication reconciliation in trauma patients and determine the challenges and strategies needed to conduct accurate medication reconciliation in this population.

## Methods

The systematic review was created to answer the question: “How effective is medication reconciliation obtained in the trauma setting?” Major databases on medical literature, including PubMed/Medline, CINAHL, EMBASE, and Cochrane Review, were searched using the keywords, Medical Subject Heading (MeSH), and search phrase “trauma OR injury, AND medication reconciliation OR med rec OR med rek, AND effectiveness OR errors OR intervention OR improvements,” as well as removing part(s) of this search phrase (i.e., “trauma OR injury, AND medication reconciliation OR med rec OR med rek”) to obtain the most relevant results. The entire available time frame of each database was included in the search. All age ranges were included.

The inclusion criteria were English language only articles that evaluated medication reconciliation in trauma patients regardless of clinical setting. Medication reconciliation in the prehospital emergency medicine services (EMS), emergency department (ED), and inpatient settings were included. The results were then screened by their title for relevance by one reviewer (JD) and were excluded if unrelated to trauma, commentaries or editorials, duplications, or did not have full publications. The remaining were screened using inclusion and exclusion criteria by two reviewers (SJ and JD). If an article could not be classified as relevant by its abstract, its full text was reviewed. The articles that met the criteria for inclusion were evaluated for the type of study (i.e., prospective, retrospective), patient populations, medication reconciliation strategies, effectiveness, challenges, and limitations. Disagreements were resolved through discussion between the reviewers. This largely had to do with deciding to leave in EMS-related papers. In case of further disagreements, our plan was to elicit one of the pharmacist co-authors to be a tiebreaker to help us decide the study’s relevance to the research question; however, this was not necessary. Preferred Reporting Items for Systematic Reviews and Meta-Analyses (PRISMA) guidelines were followed to ensure standardized search and reporting in this review [23]. We sought randomized control trials, prospective cohort studies, and case-control studies assessing accuracy of medication reconciliation in the trauma patient population and planned to combine their results and analyze them to assess for strength of the evidence. We planned to evaluate the evidence of each individual study using the following criteria: (1) < 80% follow-up; (2) > 20% missing data or missing data not at random, without proper use of missing data statistical techniques; (3) limited control of confounding; (4) more than minimal bias; (5) heterogeneity; and (6) adequacy of statistical power for studies that did not find significant differences.

## Results

**Fig. 1:**
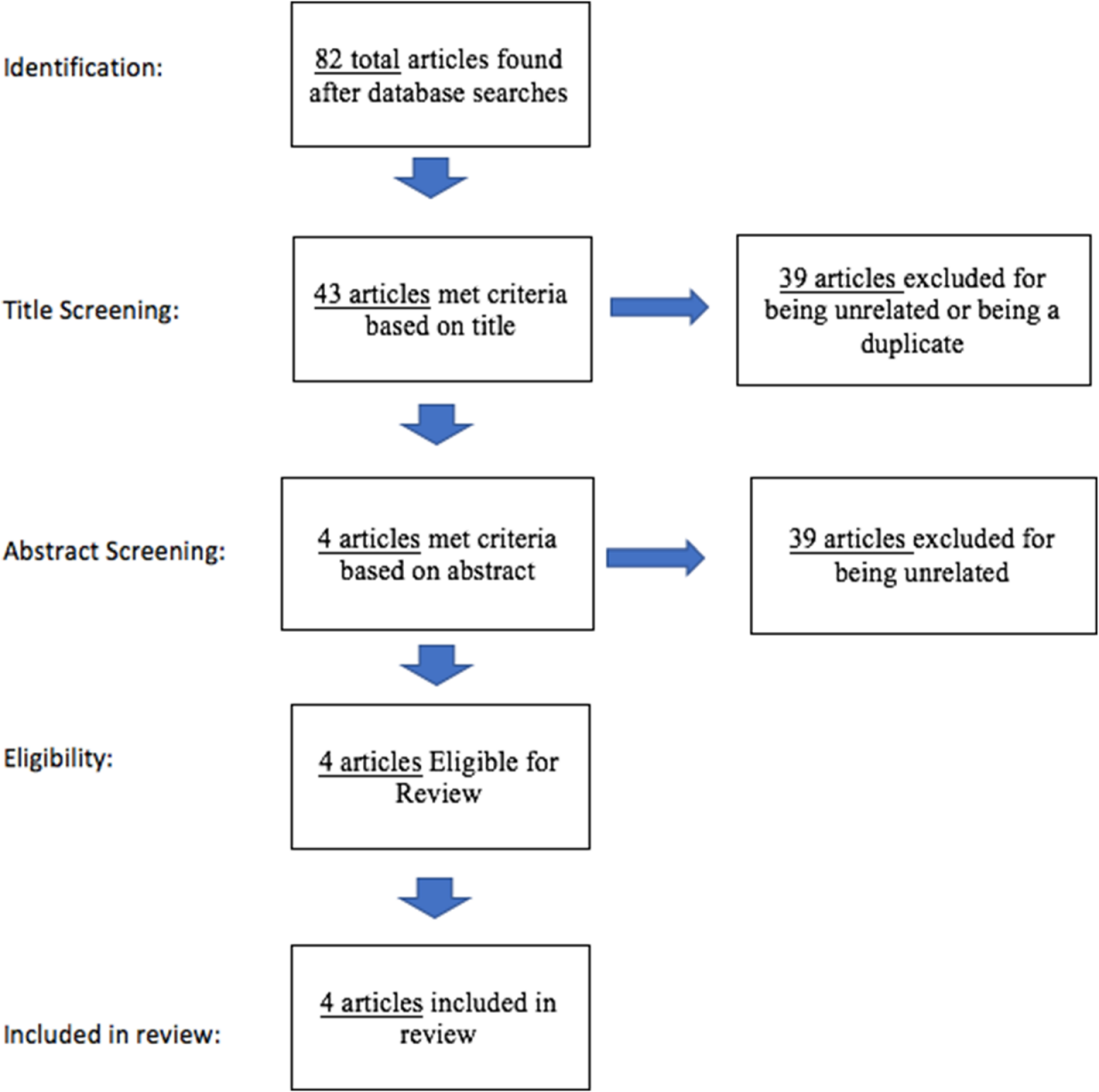
Modified PRISMA flow chart^22^ for article screening, eligibility and those included in the review

The search resulted in 82 articles. Thirty-nine were eliminated following title screening for relevance. Six duplicates were removed. Forty-three abstracts were reviewed of which four articles were eligible for the final review representing 3041 trauma patients. The results of these database searches and screening are denoted in the modified PRISMA flowchart in Fig. 1.

The search resulted in four articles that met inclusion and exclusion criteria. They had substantial differences limiting the ability to perform any meaningful comparative statistical analysis. One study involved prehospital staff, and three evaluated medication reconciliation after admission to inpatient trauma services. None were randomized control trials; two were prospective and two were retrospective. Only two of the articles looked at the accuracy or number of discrepancies between home medication lists and admission medication lists. The third study evaluated the outcomes of trauma patients who were known to specifically be on anticoagulant or antiplatelet medications, those not on these medications, and those without a known medication history. The fourth study only evaluated whether anticoagulant medications were accurately ascertained by EMS providers at the scene versus emergency department staff. Table 1 shows each study’s characteristics. No statistical analysis could be undertaken due to the limited published literature on this topic. The included studies are individually evaluated below.

**Table 1:** Summary of the articles reviewed

Study	Country of origin and type and length of study	Patient setting evaluated	Number of patients	What was evaluated?	Level of evidence and negative criteria for decreasing level of evidence	Results
S. Miller et al.	USA—prospective, 13 months	Admission to trauma service	234	MR accuracy of trauma team and admission nurse compared to pharmacist	Level IV Not RCT, no follow up required, excluded if missing data	4% overall accuracy
M. Miller et al.	Australia—retrospective, 24 months	Admission to trauma service	533	Compared patients without a medication history to those known to be currently or not currently taking (ACAP)	Level IV Not RCT, heterogeneous comparison groups	Mortality higher (*p* value = 0.004) for ACAP (24%) vs no-ACAP (11%) vs no medication history (11%); LOS, ICU LOS, vent days, disability at discharged did not differ
Pascual et al.	Spain—prospective, 1 ½ months	Admission to trauma service	164	Discrepancies in hospital medications ordered at admission when compared to home medications	Level IV Not RCT, no evaluation of heterogeneity	1(+) error(s) were found in 48.8% total; 67% admitted from ED vs. 44.8% for scheduled admission; errors of omission were the most common at 72%; risk increased by 33% for each drug taken
Nishijima et al.	USA—retrospective, 12 months	Prehospital	2110	Similarity of EMS providers MR for ACAP in head trauma compared to ED providers	Level IV Not RCT, no evaluation of heterogeneity	Similarity obtained for warfarin; not obtained for direct oral anticoagulant agents, aspirin, or other anticoagulants
**Total Patients**			**3041**			

Negative criteria for decreasing level of evidence listed in the “Methods” section

Key: *MR* medication reconciliation, *ACAP* anticoagulants and antiplatelet medications, *LOS* length of stay, *vs* versus, *ED* emergency department, *RCT* randomized control trial

Miller et al. prospectively enrolled 234 trauma patients over 13 months, from a US-based, level I trauma center, after screening 672 total patients [18]. Medication reconciliation by a clinical pharmacist was completed on average 3 days following their trauma date of admission (range 1–8 days) with a median duration of 2 days to full completion. Only 15% of medications recorded at admission were correct following the pharmacist reconciliation. The admission trauma team and nurses were inaccurate 96% of the time. Errors included medication name, strength, route, and frequency. Both the trauma team and nurses had equal accuracy when documenting type of medication, although nurses were more accurate at identifying medication strength, route, and frequency. They found that 83% of patients with an Injury Severity Score (ISS) of greater than 15 had inaccurate medication lists. Four percent of the study population had incorrectly ordered medications, and one adverse event of hypoglycemia, due to an incorrect and duplicated insulin order, was identified. This study was not a randomized control trial, no follow-up was required, and all patients with missing data were excluded. Evidence Level: IV.

Miller et al. sought to describe and compared three trauma groups from a level I trauma center in Sydney, Australia—those with medication histories currently on anticoagulants and antiplatelet medications (ACAP), those with medication histories not on ACAP, and those without medication histories [17]. Patient demographics, mechanism of injury, ISS, and clinical outcomes of these groups were compared. This was a retrospective study of 533 consecutive patients who were over 16 years of age with an ISS of greater than 12. Twenty-two percent were taking an ACAP, 57% were known to not be taking an ACAP, and 21% had no medication history on admission. ISS, injury type (blunt or penetrating), and time of day of presentation (day or night) did not affect their ability to obtain ACAP medication history. Those without a medication history had a younger median age (42 years old) when compared to the ACAP patients (82 years old). Mortality was significantly higher for patients on ACAP (24%, *p* value = 0.004) compared to those with a medication history but not on ACAP meds (11%) and those without any medication history (11%). Hospital and ICU length of stay, ventilator days, and disability at discharge did not result in different outcomes. While this was a cohort study, it has several limitations including heterogeneous populations—ACAP group was significantly older, no-ACAP group had more men, major trauma with traumatic brain injury (TBI) and isolated TBI was higher in no-ACAP group compared to others, no follow-up was required, and no information regarding missing data was included in the study. Evidence level: IV.

Pascual et al. studied the rate of medication reconciliation discrepancies in a trauma unit at a tertiary care hospital in Spain. This was a cross-sectional observational study in 164 patients over one and half months [19]. Patients were included if they were at least 18 years of age and were excluded if their medication history could not be obtained by clinical history (i.e., comatose or altered mental status) or if Spain’s National Health System pharmacy records were not available for a medication comparison. Each participant’s home medication regimen was compared to the recorded medications upon admission and throughout their stay. If a medication was added as part of the acute treatment plan, it was excluded from the evaluation. At least one medication reconciliation error was found in 48.8% of patients, and 14.4% were considered highly relevant errors, in these awake and cooperative patients who could directly report their medication history. Omission errors were the most common at 72%. The discrepancy risk increased by 33% for each additional home medication. Patients with five or more home medications had discrepancies in 67.1% of cases. The discrepancy was 33% for those on less than five medications. Patients entering the hospital through the ED had more errors compared to scheduled surgical admission patients (67% vs 44.8%, OR = 0.405 [95% CI 0.176–0.932]). This was not a randomized control study with no information on missing data or demographics. Evidence level: IV.

Nishijima et al. retrospectively analyzed 2110 patients who were 55 years or older (median age of 73 years) following head traumas throughout 1 year in California [24]. The objective was to compare EMS staff’s ability to determine anticoagulants and antiplatelet medications use by their patients compared to that of the ED and hospital staff. Five EMS services and eleven hospitals in California were included. ED and hospital staff identified 28% of patients on an ACAP compared to 16% by EMS. A kappa statistic, which evaluates the reliability or agreement between two groups, was calculated for each comparison. Similarity was achieved at greater than or equal to 0.60. For warfarin, the kappa statistic was 0.76; however, for direct oral anticoagulant agents, aspirin, and other anticoagulants, they were 0.45, 0.33, and 0.51, respectively, meaning that EMS staff were consistent with ED staff in identifying and documenting warfarin but not any of the other medications. These results were consistent across EMS agencies. There was only a slightly higher level of agreement with warfarin and aspirin for paramedics compared to non-paramedic EMS staff. Further education and training (i.e., paramedic level training) was recommended as strategies to increase medication awareness and assessment by EMS staff. This was not a randomized control study, and no statistical evaluation was done for heterogeneity. Evidence level: IV.

## Discussion

A simple PubMed search on medication reconciliation results in over 1700 papers with several in prominent journals such as BMJ and JAMA. However, based on our systematic review, there has been very few related to the trauma patient population. This is in fact the most important finding of this systematic review. Only four articles on medication reconciliation in this population were identified which suggests that the trauma community has not yet recognized the importance of medication reconciliation despite national recommendations by JCAHO. This is an area worth further research since trauma remains a leading cause of death in the USA and resulted in over $600 billion in costs in 2013 alone [25]. Furthermore, one of the strengths of this review is the systematic methodology which offers the most transparent and rigorous method of looking for existing evidence on this topic. The minimal number of papers included suggests that there is little meaningful work done on medication reconciliation in trauma despite the national mandate by the accrediting body for all healthcare organizations. Based on our results, this paper is a call to action. We urge the American College of Surgeons Committee on Trauma to consider including this aspect of trauma care in subsequent national trauma systems recommendations in line with JCAHO patient safety recommendations. Medication reconciliation in trauma is an important mechanism to keep our patients safe, and our trauma centers need to recognize the value of addressing this problem.

Our multi-disciplinary group team of surgeons, nurses, pharmacists, scientists, emergency physicians, and geriatricians have started to study medication reconciliation in trauma because we have recognized the demographic changes in the USA leading to increasing rates of elderly trauma patients who tend to be on multiple medications at baseline. This, coupled with the increasing availability of medications such as direct anticoagulants, which cannot be clinically detected at this time, makes accurate medication reconciliation in trauma vital and any errors, potentially high risk. In this systematic review, all four articles demonstrated poor accuracy of medication reconciliation in the trauma setting—4% accuracy [18] and high discrepancy rate [19]. Congruence of anticoagulant medication lists determined by EMS or hospital providers was also low [24], and medications were frequently omitted or incomplete [18, 19]. Omissions were the most common medication errors [17–19], totaling up to 72% in one article [19], consistent with the medication reconciliation reports in medicine patients [4]. Incomplete knowledge of medication usage and incorrect documentation by admitting clinicians contributed to medication reconciliation errors [18, 19]. Several other factors were attributed to these inaccuracies such as poorly informed patients, multiple pharmacy use by a single patient, medication samples obtained from physician’s offices, mail order prescriptions, Internet prescriptions, the number of medications, age of 65 or older, and emergent admission [18, 19]. These data from trauma patients are consistent with reported rates in general medical patients [3–8, 15, 16, 21, 26].

Various strategies have been reported to improve accuracy of medication reconciliation including using pharmacists and pharmacy technicians, additional training, creation of mandatory sections of the electronic medical record (EMR), or using checklists to conduct the process [4, 13, 15, 27]. Some of these interventions, such as hard stops in the EMR, however, may not apply to trauma patients as they might interfere with the emergent care of trauma patients. Since timing is important, dedicated staff can help conduct medication reconciliation. However, physician- and nurse-led medication reconciliation has poor accuracy. Pharmacist-led medication reconciliation tends to be more accurate but tends to be expensive as well. Hiring additional pharmacy technicians and pharmacists with 24-h a day, 7-day a week coverage is an expensive solution that may not apply to all facilities. Even with addition of expert staff, the process still remains very time consuming because it requires validating a patient’s medications with their various pharmacies and physicians for true accuracy, and therefore, addition of staff alone may not be enough in a trauma setting [13, 15]. Pharmacists in the Miller et al. study took on average 3 days post-trauma (range 1–8 days) to complete the medication reconciliation, with a median duration of 2 days [18]. In the general medication reconciliation literature, it took pharmacy technicians on average 23 min to provide a complete medication reconciliation in a study of over 11,000 non-trauma patients. However, these were completed in only 21% of patients before admission orders were placed [15]. If this same evaluation was applied to trauma patients, this percentage would most likely be even lower given the acuity of care. Therefore, other options should be evaluated to accurately perform medication reconciliation given time restrictions and limited resources.

Miller et al. recommended having automatic requests sent to primary care physicians upon admission of a trauma patient, designating a single member of a team to obtain medication histories, and raising awareness that omission of medication is common [17]. However, this may not be a great option in our health care environment and in the trauma setting where patients may not have dedicated primary care physicians, have physicians outside the trauma center’s network, or may not be able to communicate that information to us due to their clinical status. Nishijima et al. suggested increased education and training may improve medication reconciliation accuracy [24]. However, this would have still missed two thirds of patients on anticoagulants and antiplatelet medications since the accuracy of the hospital staff was only 28% compared to 16% by the EMS staff.

While no papers in this review addressed available electronic medical record (EMR) tools, other literature has shown that their use can improve the medication reconciliation process by identifying errors, reducing drug omission errors, comparing presenting medication lists in a side-by-side view (i.e., home medications compared to hospital medications), automatically grouping medications by therapeutic class, and effectively identifying duplicates [27–29]. EMR changes, however, often require highly skilled information technology and programming experts employed by the hospital or the EMR vendor. These changes can be costly and do not immediately support effective medication reconciliation in the trauma population. Even in settings where there are electronic systems in place, medication reconciliation is fraught with error. Furthermore, even if the prescribed medication list is known, we currently have no sense of what medications or levels of medications exist in the patient’s bloodstream or how this might affect response to treatment. It also limits our ability to treat patients since clinicians may not prescribe anticoagulant reversal agents if they do not know of patients’ prescription histories.

This review has several limitations. There were few publications on this topic with substantial variations in study design across the four publications evaluated in this review. None of the studies were randomized control trials. Two were prospective but with were major differences across the studies based on populations. These differences were health care systems (Spain, Australia, and the USA), time points of evaluation (one was prehospital, three at admission), and extent of medications assessed—two evaluated all medications, whereas the other two focused only on anticoagulants and antiplatelet medications. These differences limited any potential for comparative analysis. Numerous medications aside from anticoagulants and antiplatelet medications are considered high risk for medication errors. The Institute of Safe Medication Practices (ISMP) has generated lists of high alert medications and provided strategies to improve their use and prevent misuse [30]. These have yet to be studied in our high-risk patient population based on this review.

### Next steps

At the minimum, this review builds awareness of this important problem. Our multi-disciplinary team is currently working on additional comparative studies and investigating potential solutions which will be described in upcoming publications. One potential intervention our group is evaluating uses a plasma assay to detect prescription medications in blood much like illicit substances are currently detected. This would potentially allow results to be available rapidly to treating clinicians. Proof-of-concept testing is underway to determine if this method offers accurate, feasible results that can be fast and low-cost.

## Conclusion

The importance of medication reconciliation has been extensively studied over the past decade since The Joint Commission’s requirement for it to be a part of the entire continuum of patient care. However, there is clearly little research on medication reconciliation in our high-risk trauma patient population. The articles identified in this review show that medication reconciliation in trauma is highly inaccurate and puts patients at risk for adverse events and poor clinical outcomes. Further research is needed to evaluate medication reconciliation in the trauma setting and develop tools and techniques to improve its accuracy in our patient population.

## Abbreviations

ACAP: Anticoagulants and antiplatelet medications
ED: Emergency department
EMR: Electronic medical record
EMS: Emergency medical services
IOM: Institute of Medicine
ISMP: The Institute of Safe Medication Practices
ISS: Injury Severity Score
JCAHO: Joint Commission on Accreditation of Healthcare Organizations
Med rec or Med rek: Medication reconciliation
MeSH: Medical Subject Heading
PRISMA: Preferred Reporting Items for Systematic Reviews and Meta-Analyses
TBI: Traumatic brain injury

## References

[1] Kohn LT, Corrigan JM, Donaldson MS, Institute of Medicine Committee on Quality of Health Care in America (2000). To err is human: building a safer health system.

[2] Brennan TA, Leape LL, Laird NM, Hebert L, Localio AR, Lawthers AG (1991). Incidence of adverse events and negligence in hospitalized patients. Results of the Harvard Medical Practice Study. N Engl J Med.

[3] Bates DW (1999). Frequency, consequences and prevention of adverse drug events. J Qual Clin Pract.

[4] Kwan JL, Lo L, Sampson M, Shojania KG (2013). Medication reconciliation during transitions of care as a patient safety strategy: a systematic review. Ann Intern Med.

[5] Monte AA, Anderson P, Hoppe JA, Weinshilboum RM, Vasiliou V, Heard KJ (2015). Accuracy of electronic medical record medication reconciliation in emergency department patients. J Emerg Med..

[6] Thomsen LA, Winterstein AG, Sondergaard B, Haugbolle LS, Melander A (2007). Systematic review of the incidence and characteristics of preventable adverse drug events in ambulatory care. Ann Pharmacother.

[7] Caglar S, Henneman PL, Blank FS, Smithline HA, Henneman EA (2011). Emergency department medication lists are not accurate. J Emerg Med.

[8] Bell CM, Rahimi-Darabad P, Orner AI (2006). Discontinuity of chronic medications in patients discharged from the intensive care unit. J Gen Intern Med.

[9] Bates DW, Spell N, Cullen DJ, Burdick E, Laird N, Petersen LA (1997). The costs of adverse drug events in hospitalized patients. Adverse Drug Events Prevention Study Group. JAMA.

[10] Aspden P, Wolcott J, Bootman JL, Cronenwett LR, Institue of Medicine (2007). Preventing medication errors.

[11] Aronson J (2017). Medication reconciliation. BMJ.

[12] Catalano K (2006). JCAHO’S national patient safety goals 2006. J Perianesth Nurs.

[13] Nguyen CB, Shane R, Bell DS, Cook-Wiens G, Pevnick JM (2017). A time and motion study of pharmacists and pharmacy technicians obtaining admission medication histories. J Hosp Med.

[14] Rozich JD, Howard RJ, Justeson JM, Macken PD, Lindsay ME, Resar RK (2004). Standardization as a mechanism to improve safety in health care. Jt Comm J Qual Saf.

[15] Sadasivaiah S, Smith DE, Goldman S, Ratanawongsa N (2017). Improving best possible medication history with vulnerable patients at an urban safety net academic hospital using pharmacy technicians. BMJ Open Qual.

[16] Salvi F, Marchetti A, D'Angelo F, Boemi M, Lattanzio F, Cherubini A (2012). Adverse drug events as a cause of hospitalization in older adults. Drug Saf.

[17] Miller M, Morris R, Fisicaro N, Curtis K (2017). Epidemiology and outcomes of missing admission medication history in severe trauma: a retrospective study. Emerg Med Australas.

[18] Miller SL, Miller S, Balon J, Helling TS (2008). Medication reconciliation in a rural trauma population. Ann Emerg Med.

[19] Pascual O, Real JM, Uriarte M, Larrodé I, Alonso YM, Abad MR (2015). Evaluation of medication reconciliation in a trauma unit. Rev Esp Cir Ortop Traumatol.

[20] Pevnick JM, Shane R, Schnipper JL (2016). The problem with medication reconciliation. BMJ Qual Saf.

[21] Tam VC, Knowles SR, Cornish PL, Fine N, Marchesano R, Etchells EE (2005). Frequency, type and clinical importance of medication history errors at admission to hospital: a systematic review. CMAJ.

[22] Curtis Florence P, Tamara Haegerich P, Thomas Simon P, Chao Zhou P, Feijun Luo P. Estimated lifetime medical and work-loss costs of emergency department–treated nonfatal injuries — United States, 2013. Ctr Dis Control. 2015; Available from: https://www.cdc.gov/mmwr/preview/mmwrhtml/mm6438a5.htm. 10.15585/mmwr.mm6438a5 26421663

[23] Liberati A, Altman DG, Tetzlaff J, Mulrow C, Gøtzsche PC, Ioannidis JPA (2009). The PRISMA statement for reporting systematic reviews and meta-analyses of studies that evaluate healthcare interventions: explanation and elaboration. BMJ.

[24] Nishijima DK, Gaona S, Waechter T, Maloney R, Bair T, Blitz A (2017). Do EMS providers accurately ascertain anticoagulant and antiplatelet use in older adults with head trauma?. Prehosp Emerg Care.

[25] Centers for Disease Control and Prevention. Cost of Injury Data 2017 [Available from: https://www.cdc.gov/injury/wisqars/cost/index.html.

[26] Lesar TS, Briceland L, Stein DS (1997). Factors related to errors in medication prescribing. JAMA.

[27] Horsky J, Drucker EA, Ramelson HZ (2018). Higher accuracy of complex medication reconciliation through improved design of electronic tools. J Am Med Inform Assoc.

[28] Moro Agud M, Menendez Colino R, Mauleon Ladrero Mdel C, Ruano Encinar M, Diez Sebastian J, Villamanan Bueno E (2016). Analysis of an electronic medication reconciliation and information at discharge programme for frail elderly patients. Int J Clin Pharm.

[29] Mekonnen AB, Abebe TB, McLachlan AJ, Brien J-aE (2016). Impact of electronic medication reconciliation interventions on medication discrepancies at hospital transitions: a systematic review and meta-analysis. BMC Med Inf Decis Making.

[30] High-Alert Medications in Acute Care Settings 2014 Available from: {https://www.ismp.org/recommendations/high-alert-medications-acute-list}.

